# Salt Stress Induced Changes in the Exoproteome of the Halotolerant Bacterium *Tistlia consotensis* Deciphered by Proteogenomics

**DOI:** 10.1371/journal.pone.0135065

**Published:** 2015-08-19

**Authors:** Carolina Rubiano-Labrador, Céline Bland, Guylaine Miotello, Jean Armengaud, Sandra Baena

**Affiliations:** 1 Unidad de Saneamiento y Biotecnología Ambiental—Departamento de Biología—Pontificia Universidad Javeriana, POB 56710, Bogotá, D.C., Colombia; 2 Colombian Center for Genomics and Bioinformatics of Extreme Environments–GeBiX, Bogotá, D.C., Colombia; 3 CEA, DSV, iBEB, SBTN, Lab Biochim System Perturb, Bagnols-sur-Cèze, F-30207, France; Northeast Forestry University, CHINA

## Abstract

The ability of bacteria to adapt to external osmotic changes is fundamental for their survival. Halotolerant microorganisms, such as *Tistlia consotensis*, have to cope with continuous fluctuations in the salinity of their natural environments which require effective adaptation strategies against salt stress. Changes of extracellular protein profiles from *Tistlia consotensis* in conditions of low and high salinities were monitored by proteogenomics using a bacterial draft genome. At low salinity, we detected greater amounts of the HpnM protein which is involved in the biosynthesis of hopanoids. This may represent a novel, and previously unreported, strategy by halotolerant microorganisms to prevent the entry of water into the cell under conditions of low salinity. At high salinity, proteins associated with osmosensing, exclusion of Na^+^ and transport of compatible solutes, such as glycine betaine or proline are abundant. We also found that, probably in response to the high salt concentration, *T*. *consotensis* activated the synthesis of flagella and triggered a chemotactic response neither of which were observed at the salt concentration which is optimal for growth. Our study demonstrates that the exoproteome is an appropriate indicator of adaptive response of *T*. *consotensis* to changes in salinity because it allowed the identification of key proteins within its osmoadaptive mechanism that had not previously been detected in its cell proteome.

## Introduction

All living microorganisms are exposed to a variety of environmental parameters that define their habitats. To survive they must be able to sense environmental changes and react to with various adaptive mechanisms [1]. One of the most important environmental factors for halotolerant organisms is the salt concentration of the surrounding water while osmotic stress is a major generator of selective pressure on cells. In habitats such as saline springs the halotolerant organisms must cope with variable salinity conditions because they may have varying salt concentrations due to rains and drought. For these organisms to live under such conditions, various physiological strategies have evolved [2]. A low salinity leads to an immediate influx of water which a cell counteracts by fast efflux of small solutes thus relieving physical stress. In contrast, a high salinity leads to water efflux which is counterbalanced by an increase of compatible solutes such as proline, glutamate, glycine betaine, ectoine and trehalose [3].

*Tistlia consotensis* is a halotolerant *Alphaproteobacterium* which forms a distant phylogenetic line of descent with members of the genus *Thalassobaculum* of the family *Rhodospirillaceae* which is comprised primarily of microorganisms inhabiting marine environments [4]. *T*. *consotensis* was isolated from a terrestrial saline spring with an NaCl content of 4.5% (w/v) and which is characterized by oscillating values of chlorides (10646–15100 mg·l^-1^), electrical conductivity (29300–35150 μS·cm^-1^) and total dissolved solids (24185–41859 mg·l^-1^). This is probably related to rainfall and drought as reported previously [5]. This organism achieves optimal growth in the presence of 0.5% NaCl but can grow when salinity ranges between 0.0 and 4.0% NaCl [4]. Although *T*. *consotensis* presents a wide tolerance range of salinity, the particular characteristics of its habitat make it possible to suppose that this halotolerant bacterium is exposed to continuous changes in salt concentration. This suggests mechanisms that allow it to adapt to or to tolerate these changing conditions. Although it has been widely studied as extreme halophiles live in environments with high concentrations of salt, little is known about the mechanisms employed by halotolerant organisms to survive under changing salinity conditions.

Analysis of proteomes has been proven to be useful for prediction of structure and function of proteins, particularly those related to experimental growth conditions [6].Recently, the use of experimental proteomic data with proteogenomics has emerged as an interesting approach for improving genome annotation and for dealing with non-model organisms [7]. Interpretation of proteomic data requires a comprehensive protein sequence database. For a non-model organism, it is worthwhile to first establish the genome sequence that can then be translated into the six reading frames. The resulting protein database can be queried with experimental tandem mass spectrometry results in order to quickly establish the list of key cellular or extracellular players. Proteins may be quantified with either a labeling or non-labeling approach [6]. Although non-labeling approaches based on spectral counts are by far the most frequently used as they are simple to perform and reliable even for the analysis of extracellular components [8,9], labeling methodology is also adequate for this purpose [10]. In a previous study, we used a proteogenomic strategy to analyze changes which occurred in the cellular proteome of *T*. *consotensis* in response to different concentrations of salt [7]. This study revealed that *T*. *consotensis* combined different adaptive strategies to counteract the changes in the environmental salinity. At low salinities, the bacterium up-produces general stress proteins that contribute to proper folding and functioning of other proteins. In contrast, at high salinities *T*. *consotensis* up-produces proteins that are involved in signaling allowing it to efficiently detect changes in salinity. These proteins are also related to a large number of transport systems which are probably involved in transport and capture of compatible solutes such as glutamate [7]. Although this study identified proteins that play an important role in the osmoregulatory mechanisms of *T*. *consotensis* under hypo-osmotic and hyperosmotic conditions, information found regarding secreted proteins involved in salinity stress response was limited.

This limitation can be overcome through analysis of the using exoproteome. The exoproteome consists of all proteins secreted from extracellular and outer membranes. In the case of Gram-negative bacteria it also includes proteins from the periplasm. These proteins play roles in the organism’s survival in extreme habitats such as saline environments [8]. Even though there are few studies of exoproteome responses to salt stress, those that have been done demonstrate potential roles of altered exoproteomes in adaptive responses during salt stress. This has been demonstrated for *Burkholderia pseudomallei* which expresses metabolic enzymes, transcription/translation regulators, potential virulence factors, chaperones, phage capsid proteins, drug resistance proteins and solute transport regulator under conditions of high levels of salinity [9]. Exploring the extracellular proteome provides expanded coverage of the repertoire of proteins expressed under salt stress, and this is essential for understanding mechanisms of osmoadaptation.

Accordingly, this study aims to provide a better understanding of the adaptive response of *T*. *consotensis* to changes in salinity through analyzing the extracellular protein content when *T*. *consotensis* grows in conditions of low and high salinity. We used a proteogenomic approach to examine alterations in the exoproteome of *T*. *consotensis* under two conditions of salt stress: low salinity (0.0% NaCl, w/v) and high salinity (4.0% NaCl, w/v). We then compared these results to results obtained at optimal salinity (0.5% NaCl, w/v). Using this approach, we found that a number of secreted proteins were up-and-down-produced in response to salt stress.

## Results and Discussion

### Exoproteome of *T*. *consotensis* under salt stress

Extracellular protein expression can be seen as the physiological response of cells to a specific growth condition and also as indicators of how microorganisms interact with their environments [8]. Considering the importance of these proteins in the survival of organisms to changing environmental conditions, in this study, we analyzed the exoproteome of *T*. *consotensis* in order to complement information previously obtained from its cellular proteome and thus gain a more comprehensive view of salt stress responses in this bacterial model.

**Fig 1:**
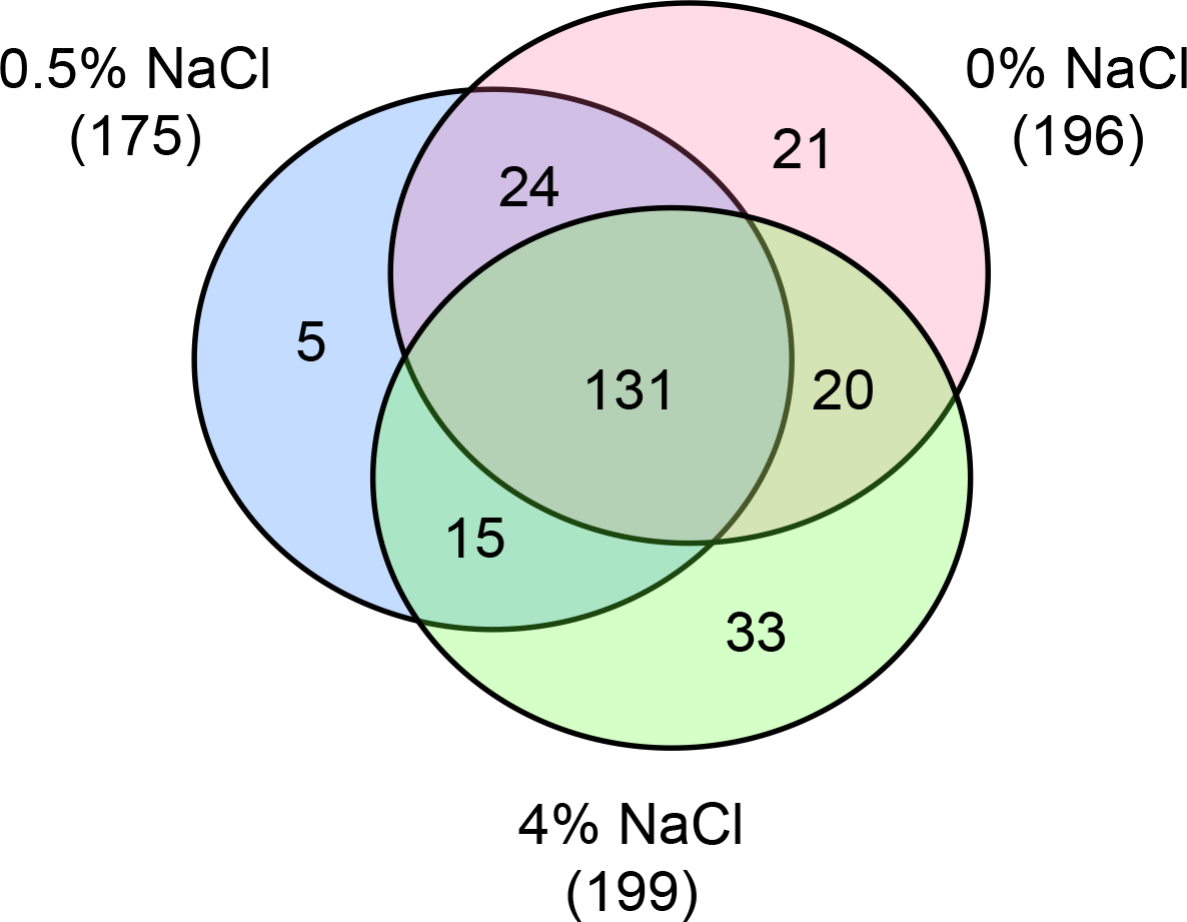
Venn diagram showing the number of extracellular proteins identified in cells grown in the three salinities. The numbers of proteins detected through identification of a least two peptides are indicated in each category.

We performed a large tandem mass spectrometry survey of the exoproteomes of four independent biological replicates for three growth conditions: 0.0%, 0.5%, and 4.0% NaCl. The twelve resulting samples were extensively analyzed with a high resolution Orbitrap mass spectrometer as analytical duplicates were recorded. The analysis of variance of the replicates for each salinity evaluated allows a determination that smaller variations occur in the total spectral counts when the cells are in the optimal growth salinity, 0.5% NaCl, (12%), than when they are in salinities of 0.0% and 4.0% NaCl in which there were variations of 14% and 18%, respectively (S1 Table). A total of 30,130 MS/MS spectra were assigned to 2,321 tryptic peptides (S2 Table). This was done with a stop-to-stop ORF database that had been constructed by six frame translation of its chimeric genome in an earlier study [7]. These peptides allowed the identification of 249 non-redundant proteins in which at least two peptides were validated by tandem mass spectrometry (S3 Table). Fig 1 shows a Venn diagram of the extracellular proteins detected within the different growth conditions. A total of 196 proteins were identified at 0.0% NaCl, 175 proteins at 0.5% NaCl and 199 proteins at 4.0% NaCl. As indicated in Fig 1, 131 proteins were common to the three conditions, which were mostly related to central metabolism processes. On the other hand, 24 proteins were common only to 0.0% and 0.5% NaCl conditions while 15 proteins were common to only 4.0% and 0.5% NaCl conditions. In both cases the majority of the proteins were again related to central metabolism. In the case of proteins common to 0.0% and 4.0% NaCl, 20 of the proteins identified were mainly involved in translation processes.

**Fig 2:**
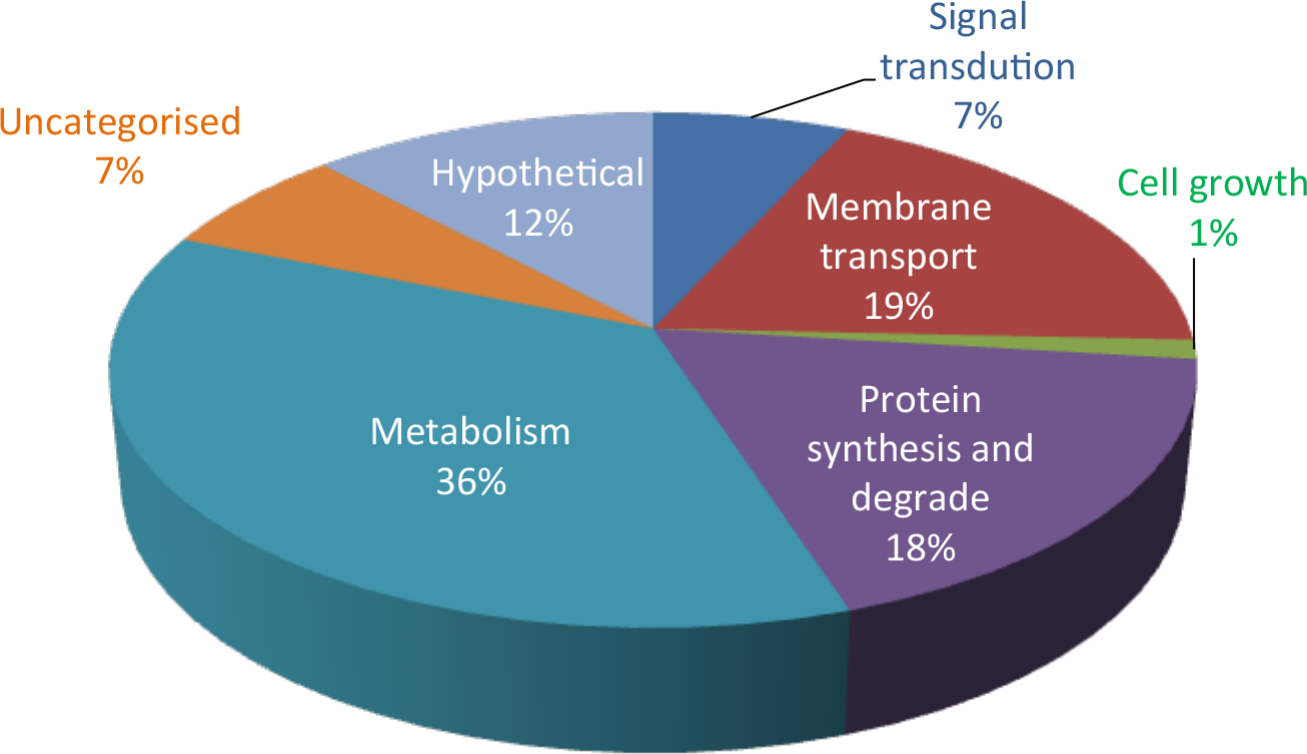
Metabolic categories for the exoproteome of *T*. *consotensis*. Functional categorization of non-redundant proteins detected in the exoproteoma of *T*. *consotensis* in the three salinities evaluated.

The distribution of the proteins detected in the three salinities based on functional categories according to the KEGG database is summarized in Fig 2. A total of 265 proteins out of the 315 proteins identified could be assigned to 40 pathways involved in *T*. *consotensis* metabolism. The proteins identified have been assigned to the following pathways: metabolism (36%), membrane transport (19%), protein synthesis and degradation (18%), signal transduction (7%), uncategorised (7%) and cell growth (1%). Hypothetical conserved proteins accounted for 12% of the total proteins identified in the exoproteome of *T*. *consotensis*.

The contaminants of cytoplasmic origins that we detected were typically proteins with intracellular functions, such as GroEL, DnaK, EF-Tu or ribosomal proteins. Their identification may indicate that cell lysis contributes intracellular material to the extracellular milieu during culturing or during the supernatant collection process. To address this issue in future studies, it is recommended that an extra step such as filtration using a 0.22 μm pore polyvinylidene fluoride (PVDF) membrane be used during the supernatant collection procedure [10]. Taking this slight contamination into account, we used the NSAF ratio to compare abundance of proteins in the secretome with their abundance in the cellular proteome, previously analyzed [7]. Proteins with a ratio (%NSAF secretome/%NSAF cellular proteome) above 1.0 were considered to be secretome specific (S4 Table). We choose this threshold value and consider that it is stringent based on previous studies. A secreted protein is expected to be more abundant in the secretome than in the cellular proteome in terms of ratio, but this depends on the different components of the secretome. The resulting global contamination rates at 0.0, 0.5 and 4.0% NaCl are shown in S5 Table: 10, 4 and 16%at 0.0, 0.5 and 4.0% NaCl, respectively.

### Signal peptide prediction of the most abundant proteins from *T*. *consotensi*s

In Gram-negative bacteria, the general secretion system directs proteins to the periplasmic space, the outer membrane or the extracellular medium. The mechanisms by which proteins are secreted or exported to different locations inside and outside of the cell frequently involve signal peptides. These N-terminal signal peptides are short chains of mostly hydrophobic amino acids that are cleavable after translocation [11]. Using the MetaLocGramN predictor we predicted subcellular localizations and the presence of signal peptides for 52 proteins found primarily in the exoproteome rather than in the intracellular proteome. Ten proteins were predicted to be extracellular, 17 proteins were predicted to be periplasmatic and 25 were predicted to be cytoplasmatic. Table 1 presents the list of the signal peptide predictions of the most abundant proteins from the *T*. *consotensis* exoproteome together with their functional annotations.

**Table 1:** Signal peptide prediction of the most abundant proteins from the *T*. *consotensis* exoproteome.

Protein ID^1^	Function annotation	gi closet protein	MetaLoGramN	PRED-TAT
**4**	TRAP transporter solute receptor TAXI family protein	153011418	Periplasmic	Sec signal
**5**	ABC-type branched-chain amino acid transport systems	83310577	Periplasmic	Tat signal
**7**	ABC-type sugar transport system, periplasmic component	90420899	Periplasmic	Sec signal
**10**	OmpAfamily protein	347757725	Periplasmic	Sec signal
**16**	Ribose ABC transporter substrate-binding protein	357029011	Periplasmic	Sec signal
**20**	Malate dehydrogenase	340027738	Cytoplasmatic	Sec signal
**21**	Extracellular solute-binding protein, family 5	91787986	Periplasmic	Sec signal
**26**	Branched-chain amino acid ABC transporter, substrate-binding periplasmic component	357428205	Periplasmic	Sec signal
**30**	Branched-chain amino acid ABC transporter substrate-binding protein	163796041	Periplasmic	Tat signal
**31**	Parvulin-like peptidyl-prolyl isomerase	288959403	Periplasmic	Sec signal
**62**	ABC transporter phosphate-binding protein	27376202	Periplasmic	Sec signal
**98**	Hopanoid biosynthesis associated membrane protein HpnM	338740576	Extracellular	Sec signal
**151**	Extracellular ligand-binding receptor	118588169	Periplasmic	Sec signal
**203**	Mu-like prophage I protein-like	218671251	Extracellular	Sec signal
**228**	Hypothetical protein SIAM614_01986	118592953	Extracellular	Sec signal
**234**	Hypothetical protein ACMV_29200	326405067	Periplasmic	Tat signal
**236**	Spermidine/putrescine ABC transporter substrate-binding protein	328544731	Extracellular	Tat signal
**246**	Hypothetical protein Rru_A2600	83593932	Periplasmic	Sec signal
**256**	NMT1/THI5 like domain-containing protein	338740779	Extracellular	Tat signal
**281**	RNA polymerase sigma-70 factor	254488172	Extracellular	Sec signal
**284**	Peptidyl-prolyl cis-transisomerase	288958015	Extracellular	Sec signal
**302**	Family 1 extracellular solute-binding protein	298290510	Periplasmic	Sec signal
**307**	Sulfate/tungstate uptake family ABC transporter, periplasmic substrate-binding protein	149928414	Extracellular	Sec signal

^1^ Protein ID refers to S3 Table.

We also used the PRED-TAT program for Sec and Tat signal peptide predictions. Most of the proteins in *T*. *consotensis* were predicted for Sec signal peptide. The Sec pathway is the main secretion system in Gram-negative bacteria. It is used for the translocation of unfolded secretory proteins and promotes the export of proteins to the periplasm [12]. Twelve proteins with Sec signal predictions were detected in the periplasmic space (Table 1). These proteins were probably translocated to periplasmatic space by the type II secretion system because proteins secreted via this pathway are synthesized with an N-terminal signal sequence which directs them to the Sec machinery in the inner membrane. After translocation across the inner membrane, the proteins appear in the periplasm where they fold into their native conformations [13]. These results indicate that the Sec pathway probably has a prominent role in protein export and secretion in *T*. *consotensis*.

### Influence of salinity on the exoproteomes of *T*. *consotensis*

For *T*. *consotensis* cells cultivated at low salinity (0.0% NaCl) we assigned a total of 10,568 MS/MS spectra in the replicates. A total of 1,574 unique peptides corresponding to 219 proteins could be detected with this pool of MS/MS spectra. In this salinity three of the proteins detected had the most significant changes (absolute fold change >1.5, *p* value <0.05). Two of these were up-produced and one was down-produced. At high salinity (4.0% NaCl) we assigned 10,813 MS/MS spectra to the replicates. A total of 1,661 unique peptides corresponding to 237 proteins could be detected with this pool of MS/MS spectra. At 4.0% NaCl, 14 of the proteins detected had the most significant changes (absolute fold change >1.5, *p* value <0.05). Thirteen were up-produced and one was found in a small amount. Table 2 shows the proteins with major changes at low and high salinities. The proteins with the most significant changes were divided into four major groups: (i) protein quality control, (ii) membrane lipid alterations for low salinity, (iii) signal transduction and (iv) compatible solute transport for high salinity. The potential roles of these proteins in response to salt stress are discussed below.

**Table 2:** Proteins with the most significant changes of *T*. *consotensis* at low and high salinity.

Protein ID^1^	Function annotation	Condition detected	Functional classification	Total spectral count	Fold change^2^	*P* Value	gi closest homologue
**98**	Hopanoid biosynthesis associated membrane protein HpnM	Low salinity	Biosynthesis metabolites secondary	47	8.1	0.04	338740576
**140**	Putative protein disulfide oxidoreductase	Low salinity	Hypothetical	28	5.1	0.03	356878066
**135**	Mu-like prophage major head subunit gpT	Low salinity	Unclassified	30	-5.4	0.00	167841676
**80**	Methyl-accepting chemotaxis protein	High salinity	Cellular Processes and Signaling	61	11.6	0.01	163793401
**81**	Putative Methyl-accepting chemotaxis sensory transducer	High salinity	Cellular Processes and Signaling	60	11.4	0.00	310825910
**72**	30S ribosomal protein S2	High salinity	Translation	64	10.1	0.04	209964520
**103**	Protease modulator	High salinity	Peptidase and post-translational modification	43	8.2	0.02	357427897
**124**	Methyl-accepting chemotaxis sensory transducer	High salinity	Cellular Processes and Signaling	36	7.8	0.02	163792755
**128**	Methyl-accepting chemotaxis protein	High salinity	Cellular Processes and Signaling	35	7.4	0.02	325293901
**61**	Fructose-bisphosphate aldolase	High salinity	Carbohydrate Metabolism	60	6.5	0.00	148259397
**146**	Trap-type mannitol/chloroaromatic compound transport system. periplasmic component	High salinity	Membrane Transport	26	6.0	0.03	304394084
**68**	Methyl-accepting chemotaxis sensory transducer	High salinity	Cellular Processes and Signaling	67	6.0	0.03	295687872
**118**	Branched chain amino acid ABC transporter periplasmic protein	High salinity	Membrane Transport	33	5.8	0.02	119899732
**153**	Methyl-accepting chemotaxis sensory transducer	High salinity	Cellular Processes and Signaling	24	5.5	0.03	328543081
**60**	Aryldialkyl phosphatase related protein	High salinity	Unclassified	72	-7.1	0.00	163798084

^1^Protein ID refers to S3 Table

^2^Fold changes represent the expression at 0.0 or 4.0% NaCl normalized to expression at 0.5%

#### Protein quality control

Under stress situations, a protein’s functional integrity is ensured through protein quality control mechanisms that include the action of molecular chaperones, protein-folding catalysts, and ATP-dependent proteases [14]. We detected high amounts of the putative protein disulfide oxidoreductase (DsbA) (5.1-fold) at low salinity. DsbA protein catalyzes disulfide bond formation into newly synthesized proteins that are translocated to the periplasm [15]. This probably indicates that DsbA protein is involved in the periplasmic folding of the Sec-dependent proteins of *T*. *consotensis*, and is translocated by the type II secretion system for translocation across the outer membrane which is the subsequent step in the secretion process. This key function of DsbA protein in processing protein translocation across the membrane explains its abundance at low salinities since formation of disulfide bonds is crucial for functioning of numerous proteins in harsh extracellular environments.

#### Membrane lipid alterations

Adaptive changes in response to salinity variations have been observed in the lipid compositions of several bacteria. These changes maintain membrane fluidity in order to preserve biological function [16]. Under conditions of low salinity, larger amounts of HpnM protein were detected (8.1-fold). HpnM protein is involved in the biosynthesis of hopanoid lipids and is member of a family of putative transporters known as "toluene tolerance protein Ttg2D”. They are in turn involved in changes in the compositions of fatty acids and phospholipids of the cell membranes of *Pseudomonas putida* which lead to increased cell membrane rigidity. These changes should be regarded as physical mechanisms that prevent solvent penetration [17].

The hopanoids are pentacyclic triterpenoids that maintain membrane stability by increasing the rigidity of the lipid matrix in a way that is similar to that of some sterols in eukaryotes and that contributes to decreasing diffusion of ions across the cell membrane [16,18]. Several studies concerning hopanoid function propose that their production may be linked to environmental and physiological changes. Some studies have proposed that hopanoids are necessary for coping with external stresses such as ethanol tolerance in *Zymomonas mobilis* [19], extreme-pH tolerance in *Alicyclobacillus acidocaldarius* [20] and *Rhodopseudomonas palustris* [21] and oxygen diffusion in *Frankia* sp. [22].

Previous studies have reported that hopanoids induce higher levels of packaging of lipids in the membrane which reduces diffusion of small molecules even including water. This has been demonstrated in *Streptomyces coelicolor* which synthesizes hopanoids to prevent water diffusion when under stress in aerial mycelium [23]. Based on our data, the detection of higher amounts of HpnM protein in *T*. *consotensis* at 0.0% NaCl probably indicates that hopanoids are involved in the alteration of the composition of the cell membrane to help minimize diffusion of water and protect the cell against the effects of the influx of water. Although to date there have been no reports about the presence of hopanoids in response to osmotic stress, the relative abundance of HpnM protein in *T*. *consotensis* could indicate a key role for hopanoids under hypo-osmotic conditions and may represent a mechanism of adaptation by *T*. *consotensis* in response to decreased in salinity which has not been previously reported. Nevertheless, additional studies are required to determine the role played by hopanoids in the cellular response of *T*. *consotensis* at low salinity.

#### Signal transduction

The evolutionary success of prokaryotes is dependent on their ability to rapidly sense and respond to changes inside and outside their cells [24]. Microorganisms have different mechanisms for sensing and responding to environmental stress, that is, ways to monitor physical and chemical changes in the external space. Signal transduction pathways transform a physical or chemical stimulus into useful signals which regulate cell functions through protein activation in the short term and through genetic regulation in the long term [3]. Some studies have shown a connection between the total number of signal transduction proteins, genome complexity (number of potential protein-coding genes) and an organism’s lifestyle [25]. Microbes with complex lifestyles and complex genomes generally encode more sophisticated and diverse regulatory systems indicating that higher degrees of complexity require higher levels of control of gene expression and cellular activity [26].

Most of the proteins up-produced by *T*. *consotensis* under conditions of high salinity were related to signal transduction proteins such as methyl-accepting chemotaxis proteins (MCPs) (11.6-fold, 7.8-fold, 7.4-fold, 6.0-fold, and 5.5-fold) and kinase CheA protein (5.0-fold). MCPs are a family of bacterial receptors that mediate chemotaxis to diverse signals by altering swimming behavior in response to changes in the concentration of attractants and repellents in the environment [27]. Kinase CheA protein is a sensor for specific extracellular environmental stimuli in bacterial two-component regulatory systems [28]. An abundance of proteins involved in signal transduction has been reported in species that are widely distributed in soils, sediments and marine and freshwater environments. Since this may be related to their ability to adapt to multiple and changing environments [29], the high number of signal transduction proteins detected at 4.0% NaCl could be related with the lifestyle of *T*. *consotensis* and could indicate an ability to efficiently sense chemical changes in external space which might allow it to adapt rapidly to changing conditions of salinity.

MCPs and kinase CheA protein which were detected at 4.0% NaCl are the two primary mechanisms by which microorganisms sense environmental signals and adopt a chemotactic response to these stimuli. Signal recognition by MCP produces a molecular stimulus that modulates CheA autophosphorylation and in turn transphosphorylation activity towards the response regulators CheY and CheB. When CheY is phosphorylated, it undergoes a conformational change which allows interaction with the flagellar motor. As a result of this interaction, the cell modulates the direction of flagellar rotation and migrates towards favorable surroundings [30].

In the case of *T*. *consotensis* under optimum growth conditions (0.5% NaCl and 30°C), Díaz-Cárdenas et al. (2010), using electron microscopy, failed to find flagella or axial filaments. However, in this study at 4.0% NaCl the flagellin protein was identified as being up-produced in the cellular proteome and was one of the most abundant proteins in the exoproteome of *T*. *consotensis* at this salinity [7]. Flagellin protein, a polymeric protein that is the principal substituent of bacterial flagella, forms helical chains around the hollow core of the flagellar filament [31].

In some halophilic organisms, such as *H*. *halophilus*, it has demonstrated that some important physiological processes are dependent on Cl^-^. These processes include germination of endospores, activation of compatible solute transporters and flagellar motility. This indicates the function of this anion in the activation of genes or proteins [32]. Roeβler& Müller (2002) analyzed the effect of Cl^-^ in *fliC* gene transcription which encodes one of the components of the flagellum. They found that the expression of this gene is stimulated in the presence of Cl^-^ [33]. This evidence may suggest that formation of flagella and chemotactic behavior by *T*. *consotensis* are activated in response to increased concentrations of Cl^-^ in the environment. This would explain the absence of flagella at optimal salinity for growth.

#### Transport system

The primary response of bacteria for counteracting high salinity involves the activation of a wide variety of specific and nonspecific transport systems. These are associated with transport of compatible solutes, accumulation of K^+^ and exclusion of Na^+^ all of which are constitutively expressed and all of which regulate the level of transcription [3]. At high salinity we detected up-production of the periplasmic component of the TRAP-type C4-dicarboxylate transport system (6.0-fold) which is involved in the exclusion of Na^+^ from the cytoplasm. The TRAP-type C4-dicarboxylate transport system is involved in the transport of C4-dicarboxylates and sugars using a gradient of Na^+^ and is important in regulation of pH homeostasis and in the sodium cycles of organisms such as *R*. *capsulatus* [34], ‘*Spiribacter salinus*’ [35] and *H*. *elongata* [36] that inhabit saline environments. The increased abundance of this type of transporter in the exoproteome of *T*. *consotensis* in response to high salinity indicates that it plays a role in the output of Na^+^ to counteract the input of Cl^-^.

We also detected up-production of a periplasmic component of branched-chain amino acid ABC transporter (5.7-fold) in response to high salinity. This protein is related to the transport of branched chain amino acids such as leucine, isoleucine and valine. In the presence of 2-oxoglutarate and pyridoxal 5-phosphate, these branched chain amino acids are converted to L-glutamate by a branched chain amino acid aminotransferase [37]. ABC branched chain amino acid (BCAA) transporters are widely distributed in various marine organisms, including *Oceanibacillus iheyensis* [37] and several marine *Salinispora*, *Bacillus* and *Roseobacter* strains [38,39]. They also account for a significant proportion of the genes observed in marine metagenomes [40]. BCAA transporters are probably an important marine adaptation because accumulated glutamate may function as a counterion for K^+^ which balances the electrical state of the cytoplasm [39]. Previous studies have reported a regulatory relationship between K^+^ and glutamate accumulation in response to osmotic stress in enteric bacteria and in the haloalkalophilic archaeon *Natronococcus occultus* [41]. Detection of larger amounts of BCAA transporter at high salinity in *T*. *consotensis* indicates that this halotolerant bacterium probably uses accumulated glutamate to maintain electrical equilibrium within the cell in response to high salt concentrations rather than as compatible solute as had previously been suggested from the analysis of its cellular proteome [7].

In relation to compatible solute transport systems, we detected an increase in the abundance of glycine betaine/L-proline binding protein (ProX) (3.1-fold) in the exoproteome of *T*. *consotensis* at 4.0% NaCl. The ProX protein is part of the ProU high-affinity transport system. This is an efficient transporter of both glycine betaine and proline betaine [42]. The ProU system is one of the most intensively characterized osmoprotectant transporters found in *E*. *coli* and *Salmonella typhimurium*. It has been reported that expression of *proVWX* structural genes strongly increases under hyper-osmotic conditions to allow increased compatible solute uptake in high osmolality environments [43]. These results indicate that, in addition to having the ability to take in glutamate as reported in the analysis of its cellular proteome [7]. *T*. *consotensis* may use glycine betaine or proline betaine as compatible solutes to counteract osmotic stress at 4.0% NaCl. Taking into account that the expression of uptake systems varies with respect to substrate specificity, mode of energetic coupling, maximum activity and regulation in response to osmotic stress [44], further studies are necessary to determine which compatible solute is preferentially expressed by *T*. *consotensis* when it is cultivated at 4.0% NaCl.

Previous studies have demonstrated that *H*. *halophilus* accumulate glutamate only transiently as a first response to salt stress but that glutamate is replaced by proline during long-term adaptation. Once the glutamate reaches a high concentration within the cell, then *pro* gene expression is stimulated by the triggering of proline production [45]. Based on our findings, we suggest that *T*. *consotensis* accumulate glutamate until a given concentration within the cell is reached. This concentration then directly leads to stimulation of ProU system expression for uptake of glycine betaine or proline betaine.

**Fig 3:**
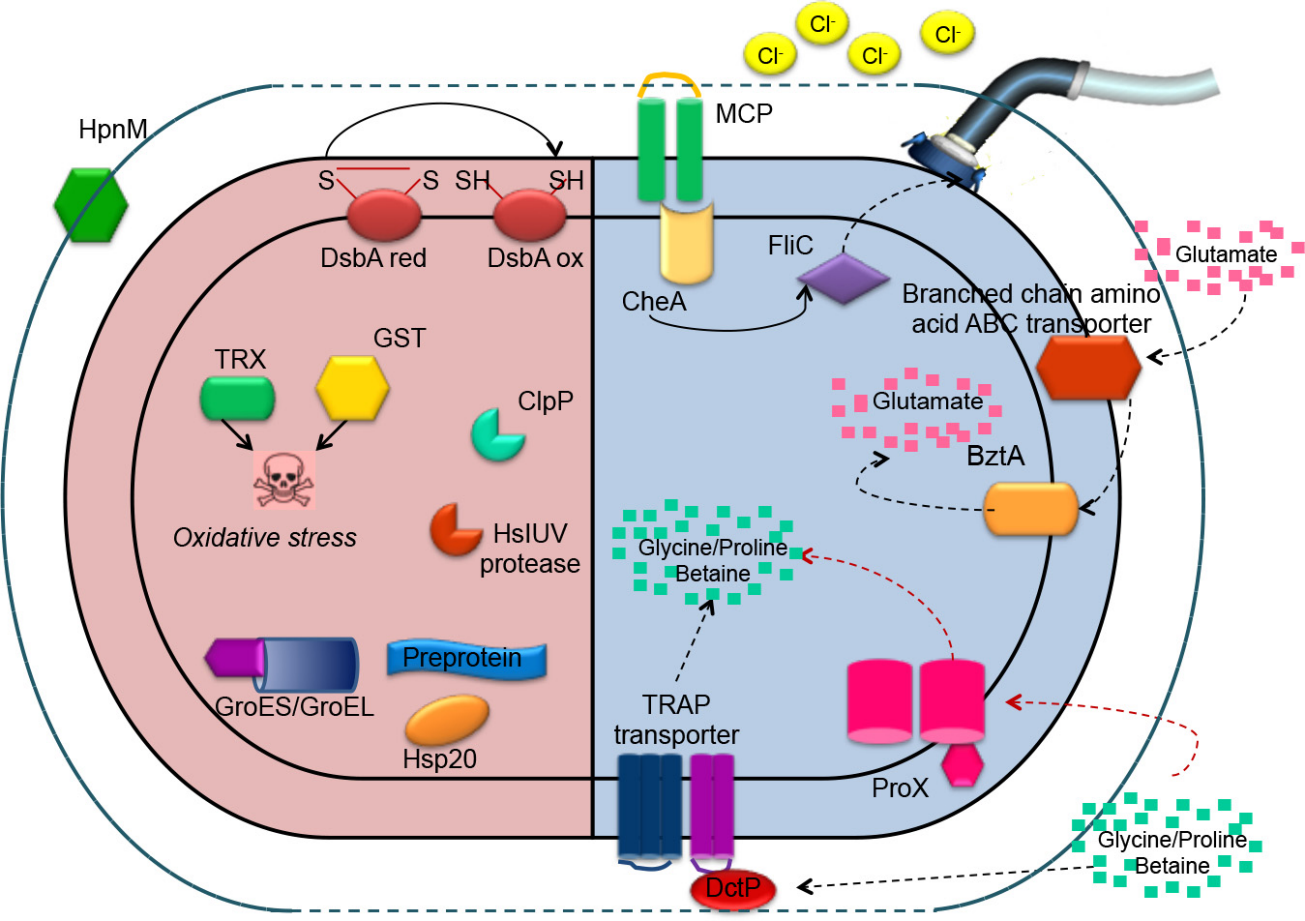
Model of the cellular processes involved in salt adaptation in *T*. *consotensis*. The pink panel shows the cellular response of *T*. *consotensis* at low salinity and the blue panel shows the cellular response of *T*. *consotensis* at high salinity. For explanations, see text.

This analysis of the exoproteome of this halotolerant bacterium provides an overview of the adaptation mechanisms it uses to cope with changing external salinities. In this paper, we have described the various responses of *T*. *consotensis* to changes of salinities in the culture medium that could not be observed by analyzing the proteome. The integration of both approaches, allowed us to summarize the adaptation mechanisms of *T*. *consotensis*. Fig 3 shows a summary of the adaptation mechanisms of *T*. *consotensis* found through detection of specific proteins under hypo- osmotic stress and hyper-osmotic stress by analysis of the exoproteome and cellular proteome of *T*. *consotensis*. General stress proteins such as chaperons, proteases and proteins associated with protection against oxidative stress are produced by *T*. *consotensis* under hypo-osmotic stress. These probably ensure proper functioning of other proteins under this condition. Also, *T*. *consotensis* secretes larger amounts of the protein HpnM which is probably involved in altering the composition of the cellular membrane to minimize the diffusion of water. In response to increased salinity, *T*. *consotensis* produces MCPs and CheA proteins responsible for efficiently sensing changes in the concentration of Cl^-^ ions. This generates a biphasic response within which the primary response is an increase in glutamate levels. This is followed by a secondary response in which uptake of compatible solutes such as glycine betaine and proline betaine increases their concentration in the cytoplasm to counteract the outflow of water under hyper-osmotic stress. This scenario shows the versatility of responses that a halotolerant organism can display and allows us to understand how its efficient responses to environmental fluctuations lead to an adaptive advantage for survival in these habitats. This study can be considered a benchmark for understanding the adaptation of organisms isolated from saline terrestrial springs.

## Materials and Methods

### Strain and growth conditions

*Tistlia consotensis*–USBA 355^T^ from our culture collection was cultured in basal medium (BM) (l^-1^) 1 g NH_4_Cl, 0.3 g K_2_HPO_4_, 0.3 g KH_2_PO_4_, 3 g MgCl_2_^.^6H_2_O, 0.1 g CaCl_2_^.^2H_2_O, 0.1 g KCl and 10 mL trace element solution (SL-10) supplemented with 2 g yeast extract, and 20 mM D-glucose as the sole carbon source. The pH of the medium was adjusted to 6.8 with 5 N NaOH [4]. *T*. *consotensis* cells were cultured at 30°C and 200 rpm in 250 ml flasks containing 100 ml of BM with 0.0, 0.5 and 4.0% (w/v) NaCl medium. For each NaCl concentration, four flasks independent (biological tetraplicates) were inoculated with *T*. *consotensis* cells previously grown on agar plate. The twelve cultures were harvested by centrifugation at 4000 rpm when the mid exponential phase (OD_600_ = 0.4) was reached.

### Protein sample preparation

Proteins were precipitated and purified from the supernatants by the addition of trichloroacetic acid (TCA) following the protocol described by [46]. Briefly, cells were removed by centrifugation at 4000 rpm for 20 min and discarded. The supernatants collected (40 ml) were precipitated by treating with 50% (v/v) TCA (Sigma- Aldrich) in ice for 10 min. After, the precipitate was collected by centrifugation at 4000 rpm for 15 min at 4°C. The resulting pellets were resuspended in 30 μl of lithium dodecyl sulphate-β-mercaptoethanol protein gel sample buffer (LDS sample buffer) (Invitrogen) and incubated at 99°C for 5 min prior to SDS-PAGE.

### Trypsin in-gel proteolysis and nanoLC-MS/MS analysis

The samples of exoproteome (20 μl) were resolved by denaturing dodecyl sulfate polyacrylamide gel electrophoresis (SDS-PAGE) on 4% to 12% gradient NuPAGE (Invitrogen) gels with a migration of 5 min at 200 V. MES (Invitrogen) was used as running buffer. Gels were stained with Simply Blue Safe Stain (Invitrogen). Each gel lane was divided into two fractions and cut. Polyacryamide gel bands were processed for *in-gel proteolysis* with trypsin (Roche) following the ProteaseMax protocol (Promega) as previously described [47].

### NanoLC-MS/MS analysis

Each biological replicates was analyzed by duplicate resulting 24NanoLC-MS/MS experiments (experimental replicates). The 24 resulting peptide mixtures were performed using an LTQ-Orbitrap XL hybrid mass spectrometer (ThermoFisher) coupled to an UltiMate 3000 LC system (Dionex-LC Packings) in conditions similar to those previously described [48,49]. Peptide mixtures (10 μl) were loaded and desalted on-line in a reverse phase precolumn (Acclaim PepMap 100 C18, 5μm bead size, 100 Å pore size, 5mm x 300μm) from LC Packings at a flow rate of 0.3 μl/min with a gradient of CH_3_CN/0.1% formic acid prior to injection into the ion trap mass spectrometer. Peptides were resolved using a 90 min gradient from 5% to 60% of solvent B (0.1% HCOOH/80% CH_3_CN). Solvent A was 0.1% HCOOH/100% H_2_O. Full-scan mass spectra were measured from *m/z* 300 to 1,700 with the LTQ-Orbitrap XL mass spectrometer in data-dependent mode using the TOP7 strategy. In brief, a scan cycle was initiated with a full scan of high mass accuracy in the Orbitrap. This was followed by MS/MS scans in the linear ion trap of the seven most abundant precursor ions, with dynamic exclusion of previously selected ions.

### Genome sequencing of *T*. *consotensis* and 6-frame ORF database

Genomic DNA extraction and sequencing of *T*. *consotensis* cells was performed as described by Rubiano-Labrador et al. (2014). Briefly, the DNA was fragmented and a 454-library with an average 8-kb insert size was constructed and sequenced on the Roche 454 GS FLX+ System (Titanium XL+ sequencing chemistry) by the GATC company. A total of 171,055 sequencing reads were recorded which resulted in 59,754,518 bases read. De novo assembling with the 454 Newbler assembler tool resulted in 2377 contigs (5,701,113 nt, 161,098 reads used) for the *T*. *consotensis* genome (10.4× coverage). This Whole Genome Shotgun project has been deposited at DDBJ/EMBL/GenBank under the accession numbers CBKU010000001-CBKU010002377 and is available at the European Nucleotide Archive (http://www.ebi.ac.uk/ena/data/view/CBKU010000001-CBKU010002377). The 2377 contigs sequences were concatenated end to end (from the largest to the shortest) to create a virtual continuous genome sequence. This artificial chimeric sequence was translated into the six reading frames using Bioedit version 7.1.3 software which resulted in ORF polypeptide sequences (from stop to stop). The resulting ORF database comprised 52,246 putative polypeptide sequences, totaling 9,292,134 amino acids with an average of 178 amino acids per putative polypeptide. Proteomic data and proteogenomic procedures were then used to discriminate between false, correct but undetected, and correct and detected protein sequences as described earlier [48].

### MS/MS database search

The recorded MS/MS spectra of one fraction of each replicate were merged before a further search against our home-made ORF database was done. First, peak lists were generated with MASCOT DAEMON version 2.3.2 from Matrix Science using the extract_msn.exe data import filter from the Xcalibur FT package (version 2.0.7) proposed by ThermoFisher. Data import filter options were set as previously described at 400 (minimum mass), 5,000 (maximum mass), 0 (grouping tolerance), 0 (intermediate scans), and 1,000 (threshold) [50]. We performed a search for the MS/MS spectra in the ORF database with the following parameters: tryptic peptides with a maximum of 2 missed cleavages during proteolytic digestion, a mass tolerance of 5 ppm on the parent ion, and MS/MS of 0.5 Da; fixed modification for carbamidomethylated Cys (+57.0215); and variable modification for oxidized Met (+15.9949). All peptide matches with the ORF database with a peptide score above the query threshold of *p*< 0.05 and rank 1 were parsed using the IRMa 1.28.0 [51]. Using a decoy database the false-positive rate for peptide identification with these parameters was estimated at below 0.5%. MS/MS spectra assigned to several loci were systematically removed. A total of 80,542 MS/MS spectra were recorded during the 24 nanoLC-MS/MS runs. Four biological repeats were done for each of the three growth media and the two analytical replicates. A protein was considered validated when at least two different peptides of that protein were detected in the same experiment. Using a reverse decoy database false-positive identification of proteins with these parameters was estimated at below 0.1%. The six reading frame ORF database from genome sequencing of *T*. *consotensis* previously constructed was directly used for assigning tandem mass spectrometry spectra.

### Comparison of protein abundance with PatternLab

The number of MS/MS spectra per protein (spectral counts) was determined for the different replicates under each growth condition. A unique biological replicate, 0.5% NaCl, was considered to be an outlier because of its significantly low number of assigned spectra. The salinity of 0.5%NaCl was used as a control condition for the comparative analysis of the abundance of the detected proteins. Protein abundances were first compared between 0.0% NaCl medium (8 replicates) and the reference medium of 0.5% NaCl (6 replicates), and between 4.0% NaCl medium (8 replicates) and the reference medium of 0.5% NaCl (6 replicates). A list of non-redundant proteins detected among the corresponding datasets was established for each of these two comparisons. The total spectral count of each polypeptide was used to rank the proteins detected from highest to lowest. The statistical variation of proteins among the replicates under the two specific growth conditions compared was calculated using the T-Fold option of PatternLab 2.0 [52]. This module allows normalization of spectral count datasets, calculating average fold changes with the t-test, and estimating the resulting theoretical false discovery rate. Stringent parameters were used for this analysis: minimum fold change of 1.5, minimum *p*-value of 0.05 and BH-FDR Alfa of 0.15. The normalized spectral abundance factor (NSAF) for each protein was calculated as described [50].

### Protein and nucleic sequence analysis

A BLASTp search was performed at the NCBI website facilities (www.blast.ncbi.nlm.nih.gov) using the non-redundant protein sequences and default parameters. The functional annotations of all proteins identified in this study were further confirmed using the KEGG database (www.genome.jp/kegg/). The genomic contexts of specific loci were inspected using the GenoMapper tool [53]. For the predictions of signal peptides, proteins identified exclusively in the exoproteome of *T*. *consotensis*, *i*.*e*. which were not present in the cellular proteome were manually re-annotated in terms of sequence structure through inspection of the corresponding stop-to-stop open reading frame and blast results. The sequences obtained were exported into a FASTA file for evaluation by the MetaLocGramN facilities (http://genesilico.pl/MetaLocGramN/) [54], SecretomeP 2.0 with settings for Gram-negative bacteria (http://www.cbs.dtu.dk/services/SecretomeP/) [55]and PRED-TAT (http://www.compgen.org/tools/PRED-TAT) [56]. On the basis of the NSAF percentage, a ratio was calculated for each protein identified in the proteomic dataset. This was used to compare their abundance in the secretome with their abundance in the previously analyzed global cellular proteome [7]. Proteins with a ratio (%NSAF secretome/%NSAF cellular proteome) above 1.0 were considered to be secretome specific.

## Supporting Information

S1 Table Analysis of variation among the replicates in each salinity. (XLSX)

S2 Table List of MS/MS spectra assigned to peptides from *Tistlia consotensis* exoproteomes. (XLSX)

S3 Table List of proteins from *Tistlia consotensis* exoproteome. (XLSX)

S4 Table NSAF ratio for all proteins detected. (XLSX)

S5 Table Rate of contamination with intracellular proteins. (XLSX)
